# Geminal Charge-Assisted Tetrel Bonds in Bis-Pyridinium Methylene Salts

**DOI:** 10.1021/acs.cgd.2c01386

**Published:** 2023-02-14

**Authors:** Miriam Calabrese, Andrea Pizzi, Andrea Daolio, Maurizio Ursini, Antonio Frontera, Nicola Demitri, Carsten Lenczyk, Jakub Wojciechowski, Giuseppe Resnati

**Affiliations:** † NFMLab, Department of Chemistry, Materials, and Chemical Engineering “Giulio Natta”, Politecnico di Milano, 20133 Milano, Italy; ‡ Department of Chemistry, Universitat de les Illes Balears, Crta. de Valldemossa km 7.5, 07122 Palma de Mallorca, Baleares, Spain; § Elettra Sincrotrone Trieste, S.S. 14 Km 163.5 in Area Science Park, Basovizza, 34149 Trieste, Italy; ∥ Bruker AXS GmbH, Oestliche Rheinbrueckenstr. 49, 76187 Karlsruhe, Germany; ⊥ Rigaku Europe SE, Hugenottenallee 167. Neu-Isenburg 63263, Germany

## Abstract

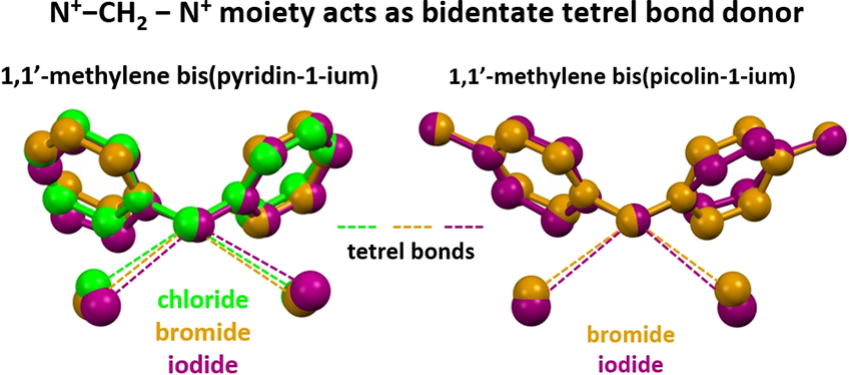

C(sp^3^) atoms are known to act as electrophilic sites in self-assembly processes, and in all cases reported till now, they form only one interaction with nucleophiles; that is, they function as monodentate tetrel bond donors. This manuscript reports experimental (X-ray structural analysis) and theoretical evidence (DFT calculations), proving that the methylene carbon in bis-pyridinium methylene salts establishes two short and directional C(sp^3^)···anion interactions; that is, they function as bidentate tetrel bond donors.

## Abstract 2

Experimental and computational evidence proved that the C(sp^3^) in 1,1′-methylene bis(pyridin-1-ium) and picolin-1-ium salts forms two geminal tetrel bonds strong enough to affect the packings of these salts.

## Introduction

The electrophilic behavior of some C(sp^2^) and C(sp) carbon atoms (e.g., of carbonyl and cyano groups) is well known and widely exploited in synthetic chemistry. After the seminal papers of Buergi and Dunitz,^1^ this behavior has become a central topic also in supramolecular chemistry.^2^ Similarly, the electrophilic behavior of C(sp^3^) carbon atoms is an established feature in synthetic processes (e.g., S_N_2 reactions), but its importance in recognition and self-assembly processes has been recognized only recently.^3^ The physical origin of this behavior is the presence of regions of depleted electron density (σ-holes)^4^ on C(sp^3^) atoms just opposite to the covalent bonds formed by carbon. If the atom/group bonded to carbon is a strong electron-withdrawing residue, the electrostatic potential at the σ-holes is positive and attractive interactions (tetrel bonds (TtBs))^5^ can be formed with regions, in the same or nearby molecules, having a negative electrostatic potential. The ubiquitous presence of C(sp^3^) carbon atoms in organic compounds rapidly drew major attention on thus formed C(sp^3^)···nucleophile/Lewis base interactions in fields as diverse as physical organic chemistry,^6^ structural biology,^7^ and crystal engineering.^8^ In all reported cases, carbon forms one such interaction; that is, it acts as a monodentate TtB donor.

We reasoned that the presence of two particularly strongly electron-withdrawing groups bonded to carbon would generate two strongly positive σ-holes, possibly enabling carbon to function as a bidentate TtB donor. The pyridinium group is a very strong electron withdrawing residue, and we decided to focus our attention on 1,1′-methylene bis(pyridin-1-ium) moieties due to their frequent presence in systems as interesting as catenanes, rotaxanes, knots, and mechanically bonded structures.^9^

**Scheme 1:**
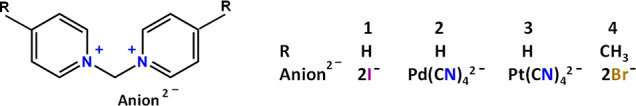
Molecular Structures of Studied Bis-Pyridium and Bis-Picolinium Methylene Salts

Here, we report experimental (X-ray structural analysis) and theoretical evidence (DFT calculations), proving that the methylene carbon of the four 1,1′-methylene bis(pyridin-1-ium) and bis(picolin-1-ium) salts **1**–**4** (Scheme 1) forms two short^10^ and directional C···I/Br/N contacts; that is, it indeed acts as a bidentate TtB donor.

## Experimental Section

### Materials and Methods

Starting materials and solvents were purchased from commercial suppliers (e.g., Sigma-Aldrich) and used without any further purification for synthesis and crystallization.

IR spectra were obtained using a Nicolet NEXUS FT-IR spectrometer equipped with a UATR unit. ^1^H and ^13^C NMR spectra were recorded at ambient temperature on a Bruker AV-400 spectrometer. All chemical shifts in the Supporting Information are given in ppm. D_2_O-d_2_ was used as a solvent. Single-crystal X-ray data for the studied compounds were collected at the XRD2 beamline of Elettra Synchrotron, Trieste (**1**), using a Bruker D8 VENTURE diffractometer (**2**), a XtaLAB Synergy diffractometer (**3**), and a Bruker SMART APEX II CCD diffractometer (**4**). Technical details regarding instruments, essential crystals, and refinement data are reported in detail in the Supporting Information.

CIF files containing crystallographic data can be obtained free of charge from the Cambridge Crystallographic Data Centre.

For the energetic calculations and NBO analysis, the GAUSSIAN-16 program was used while QTAIM analysis was performed using the AMAII program (see the Supporting Information for more details).

## Results and Discussion

In the four examined salts, it can be expected that the by far strongest force determining the crystal packing is the cation–anion electrostatic attraction. Moreover, a dense network of hydrogen bonds (HBs) connects the anion and the electropositive hydrogen atoms of the cation. Arguably, this network seriously affects the overall crystal packing. For instance, *ortho* pyridine hydrogen atoms give remarkably short contacts (e.g., a C–H···I separation as short as 287.7 pm and a C–H···N separation as short as 237.0 pm are present in **1** and **2**, respectively, both values corresponding to a normalized contact Nc^11^ of 0.85). Quite close HBs are formed also by hydrogen atoms in the *meta* and *para* positions of the pyridine ring and by those of the methylene group (e.g., such C–H···N separations as short as 237.4, 246.9, and 241.5 are present in **2** and **3**, corresponding to Nc values being 0.85, 0.89, and 0.86 in the order).

Despite the severe structural constrains caused by attractive forces mentioned above, the electrophilicity of the methylene carbon is robust enough to determine the presence of two short and directional C(sp^3^)···anion TtBs and neutral trimeric units are well-defined supramolecular motifs in the four salts.

The two symmetry-related C···I separations in **1** are 363.9 pm (Nc = 0.94), and the N–C···I angles are 168.51°. The two C···N separations in tetracyanido platinate **3** are 317.7 (Nc = 0.96) and 325.6 (Nc = 0.98), and the N–C···N angles are 169.21° and 177.47°. Very similar C···N distances and N–C···N angles are observed in tetracyanido palladate **2**. In all these interactions, the nucleophilic atom of the anion gets close to carbon on the approximate elongation of the N–C covalent bonds; that is, according to the expected directionality of a TtB.^8^ This supports the rationalization of the C···I/N short contacts as charge-assisted TtBs.^12,13^

**Figure 1:**
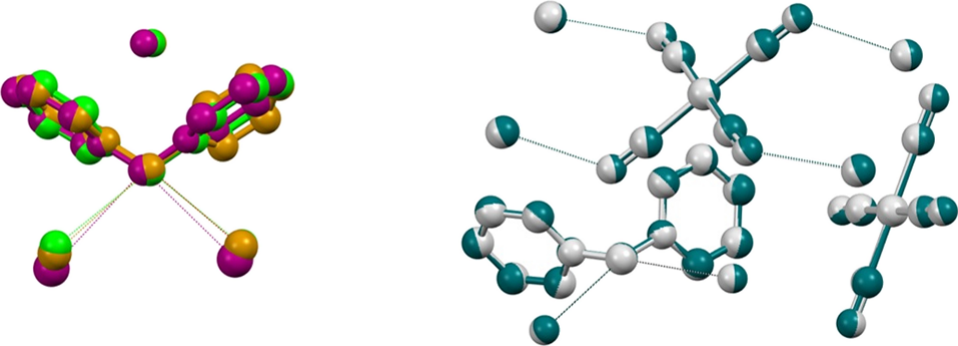
Overlays (Mercury, 2022.1.0) of the crystal unit cells of: (left) hydrated bis-pyridinium methylene chloride (green, refcode NUQXED), bromide (brown, refcode RAHZUX), and iodide (violet, **1**), water molecule is top mid; (right) bis-pyridinium methylene tetracyanido palladate (teal, **2**) and platinate (gray, **3**). In both overlays, hydrogen atoms have been omitted for clarity; only the TtBs involving the C(sp^3^) atoms are reported as a dotted line in the color of the corresponding units.

**Figure 2:**
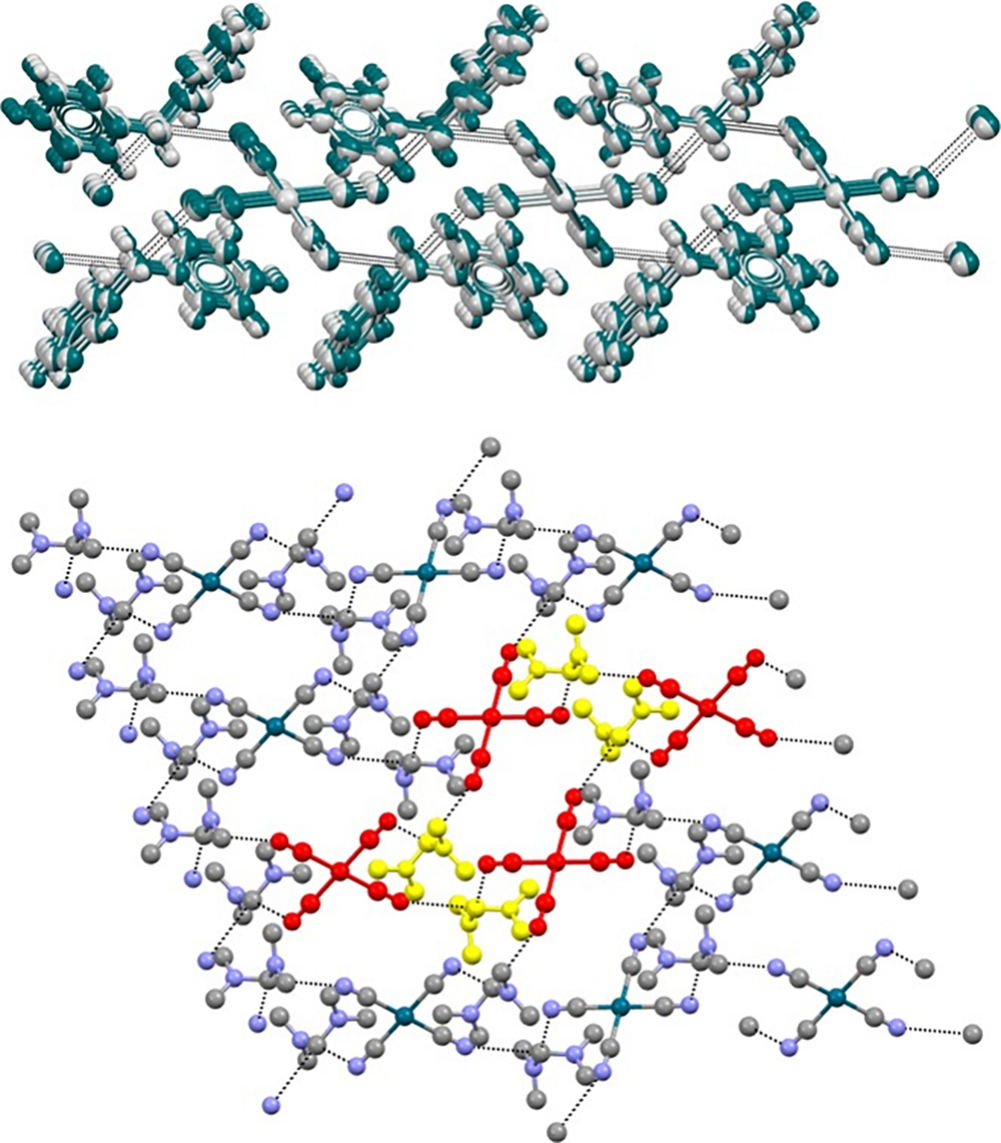
Top: partial view (Mercury, 2022.1.0) of the overlaid crystal packing of bis-pyridinium methylene tetracyanido palladate (teal color, **2**) and platinate (gray color, **3**). The view (approximately along the *b* axis) evidences the layer formed by the tetrel bonded (4,4) net. TtBs are teal/gray dotted lines. Bottom: partial view (along the *a* axis) of the crystal packing of palladate **2** evidencing the tetrel bonded (4,4) net. For the sake of clarity, hydrogen atoms and meta and para carbon atoms of pyridinium rings have been omitted. TtBs are black dotted lines. Color codes: gray, carbon; blue, nitrogen; teal, palladium. Atoms of the “nodes” and the “spacers” of one topological unit of the (4,4) networks are in red and yellow, respectively.

When different halide anions interact with a given electrophilic partner, they can give rise to adducts with the same topology.^14,15^ This behavior is probably related to the fact that they are spherical anions and do not have major directional preference in their coordination spheres so that the adopted interaction patterns are dictated by the electrophile. In other cases, different halides interacting with the same partner, e.g., via HBs^16,17^ or HaBs,^18^ drive the formation of different supramolecular arrays. In these cases, the different nucleophilicity and size of different halides seem to prevail over other factors. Similar to the bis-pyridinium methylene iodide **1**, the chloride^19,20^ and bromide^21^ analogues crystallize as monohydrates, adopt the uncommon Fdd2 space group, and form two symmetry-related C···Cl/Br TtBs. These TtBs drive the formation of three quite similar supramolecular trimers (Figure 1, left), indicating that the TtB donor ability of the C(sp^3^) in the bis-pyridinium methylene cation is robust enough to overcome the possibly different packing preferences resulting from the different nucleophilicity and size of the three halides. Analogously, tetracyanido palladate and platinate anions coupled with the same cation may adopt the same^22^ or different^23^ crystal packings. Salts **2** and **3** both crystallize in the P2_1_/c space group and have strictly similar unit cells (Figure 1, right). In both structures, CH_2_ carbon atoms function as bidentate TtB donors, one tetracyanido metallate anions is not involved in the formation of this type of interactions while the other acts as a tetradentate TtB acceptor. Resulting TtBs form very similar supramolecular patterns, which, from the topological point of view, are (4,4) networks wherein anions and bis-pyridinium methylene cations function as nodes and node spacers (Figure 2).

The structure of the bis-picolinium methylene bromide **4**, similar to that of bis-picolinium methylene iodide,^24^ parallels structure **1** as far as the network of HBs and, most importantly, TtBs, is concerned. Specifically, four symmetry-related and short contacts between bromide anions and *ortho* pyridine hydrogen atoms are present (277.8 and 278.6 pm, Nc = 0.88). Two symmetry-related N–C···Br TtBs are formed by the CH_2_ group and assemble well-defined neutral trimeric motifs, wherein C···Br separations and N–C···Br angles are 347.3 pm (Nc = 0.95) and 170.06°.

**Figure 3:**
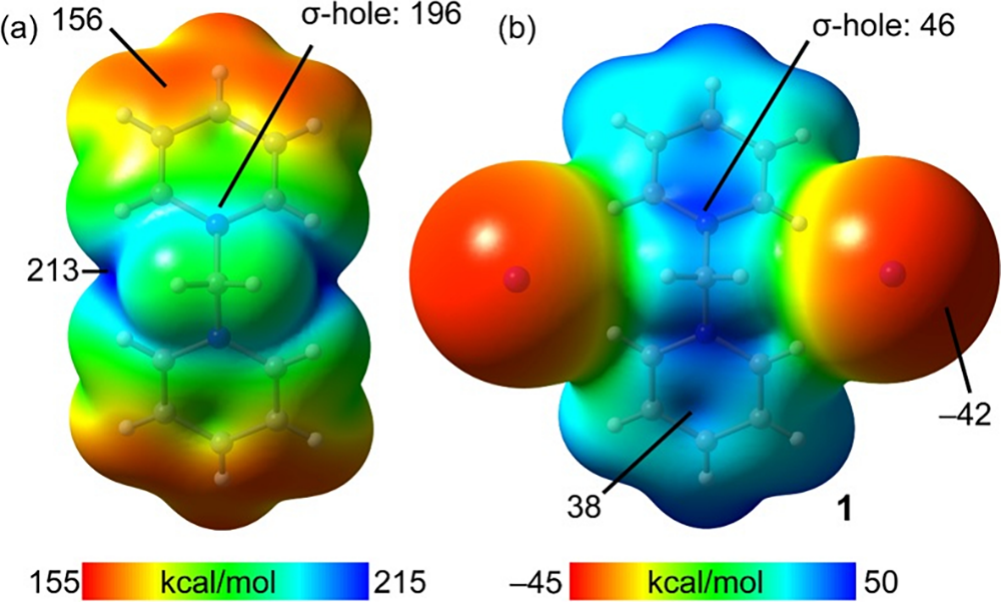
MEP surface plots of isolated bis-pyridinium methylene dication (a) and neutral trimeric unit of **1** (b) at the PBE0-D3/def2-TZVP level of theory. The values at selected points of the surfaces are given in kcal/mol. Isovalue 0.001 a.u.

Calculations (see the ESI for details) were performed to investigate the existence of σ-holes at the C(sp^3^) atom of the bis-pyridinium methylene cation opposite to both C(sp^3^)–N^+^ bonds and to rationalize as TtBs the short C···N/Br/I contacts observed in crystals **1**–**4**. The MEP surface of the bis-pyridinium methylene moiety is given in Figure 3, showing that the MEP is positive in the entire surface, as expected for a dicationic molecule. The global MEP maxima are observed at the two regions where the *ortho* H-atoms of both pyridinium rings converge (Figure 3a, 213 kcal/mol).

**Figure 4:**
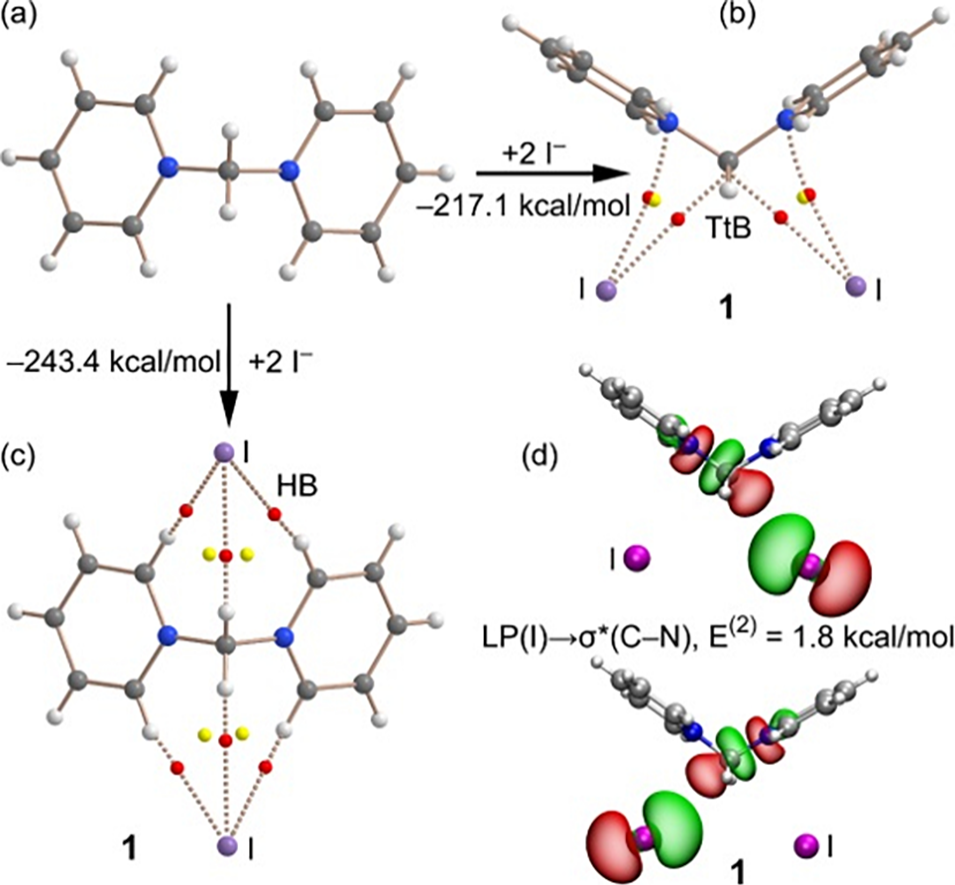
(a) Representation of bis-pyridinium methylene dication. QTAIM analysis of the TtB (b) and HB (c) binding modes of **1**. Bond and ring CPs are red and yellow spheres. Bond paths are dashed lines. Only intermolecular interactions are represented. (d) Plots of the donor and acceptor NBOs and the second-order perturbation energy.

There are also two regions of locally the most positive MEP opposite to the C–N bonds, confirming the existence of the σ-holes. Agreeably with the MEP analysis, in the solid state of compounds **1**–**4**, two anions are indeed located at the maxima of dication MEP (Figures 3 and 4). We have also computed the MEP surface of a bis-pyridinium iodide unit in the geometry adopted in crystal **1** (Figure 3b). It is observed that MEP values are considerably reduced compared to the isolated dication due to the neutral nature of the unit. Importantly, MEP values are maxima at the two regions opposite to the C–N bonds (+46 kcal/mol, σ-holes). The MEP values are also positive over the pyridinium ring centers (+38 kcal/mol), thus suitable to establish anion−π interactions.

We analyzed compound **1** both energetically and topologically using the quantum theory of atoms-in-molecules (QTAIM).^25^ The results are shown in Figure 4, where we have analyzed the two preferred anion binding modes, driven by HB and TtB, of the bis-pyridinium methylene salt (Figure 4a–c). Binding energies of both modes are very large and negative (−243.4 kcal/mol for the HBs and −217.1 kcal/mol for the TtBs) due to the cation–anion attraction in the ion pair. The HB-based binding is stronger in line with the MEP analysis. The QTAIM analysis of this assembly reveals that each anion forms three H-bonds with the CH groups of the cationic moiety, each characterized by a critical bond (CP, red sphere) and bond path (dashed line). The binding mode is further characterized by two ring critical points (yellow spheres) due to the formation of two supramolecular rings. For the TtB complex, each anion is indeed connected to the C(sp^3^) by a bond CP and bond path, thus confirming the formation of the C(sp^3^)···I^–^ TtBs. The anions are also connected to the N atom of the pyridinium rings, thus suggesting the formation of an anion−π interaction. The value of the electron density at the CP characterizing the TtB is slightly greater (ρ = 0.0079 a.u.) than that characterizing the anion−π interaction (ρ = 0.0074 a.u.), thus indicating that the TtB is stronger than the N···I^–^ bond.^26^

Charge transfer occurs in TtBs, where a filled molecular orbital at the electron donor (usually a lone pair (LP)) donates charge to the antibonding σ* orbital of the σ-hole donor. We studied if such phenomenon occurs in the TtB observed in compound **1** by using the natural bond orbital (NBO) analysis.^27^ Gratifyingly, we observed a donation of electron density from an LP (for each iodide anion) to the antibonding C–N orbital with a total stabilization energy of *E*^(2)^ = 1.8 kcal/mol (Figure 4d). Although this contribution is much smaller than the ion–pair attraction (Coulombic force), it is important to ratify the σ-hole TtB nature of the C(sp^3^)···I^–^ contacts.

**Figure 5:**
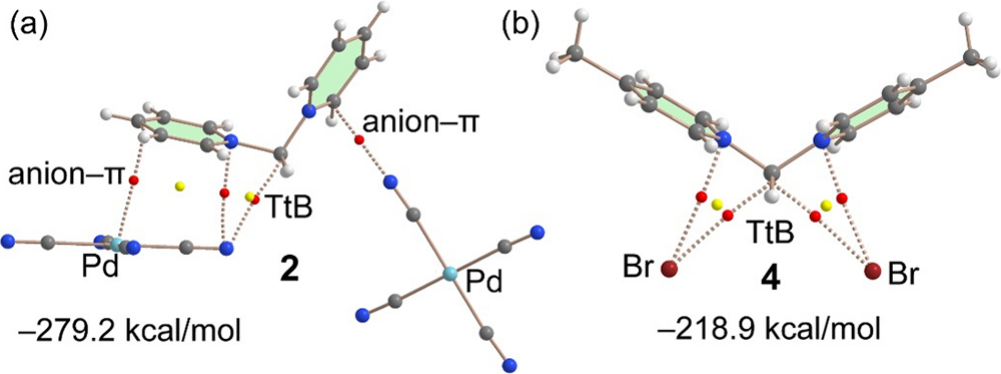
QTAIM analyses of **2** (a) and **4** (b). Bond and ring CPs are represented as red and yellow spheres, respectively. Bond paths as dashed lines. Only intermolecular interactions are represented.

The QTAIM analyses of the TtB interactions in compounds **2** (also as model of **3**) and **4** are given in Figure 5 (see Figure S5 for compound **3**). It is observed that for compound **2**, the QTAIM analysis shows that only one tetracyanido palladate anion is connected to the C(sp^3^) atom by a bond CP and bond path. The other anion is connected to the aromatic ring forming an anion−π interaction. Although the QTAIM method confirms the TtB only for the closest anion, the noncovalent interaction plot (NCIplot)^28^ (a convenient computational tool to reveal interactions in real space) suggests the existence of the TtB also for the anion with the longest C···N distance. Indeed, a small reduced density gradient (RDG) isosurface appears between the N atom of the cyanido ligand (Figure S5) and the C(sp^3^) atom, thus confirming the TtB.

In case of compound **4** (Figure 5b), the distribution of bond CPs and bond paths is almost identical to that of compound **1**, confirming the formation of both TtBs and the ancillary anion−π interactions. Moreover, the interaction energy obtained for **4** (−218.9 kcal/mol) is very similar to that of **1**. In contrast, the binding energy for compound **2** is significantly greater (−279.2 kcal/mol), likely due to the orientation of one of the tetracyanido palladates that establishes a strong anion−π interaction (see also RDG isosurface in Figure S5).

**Figure 6:**
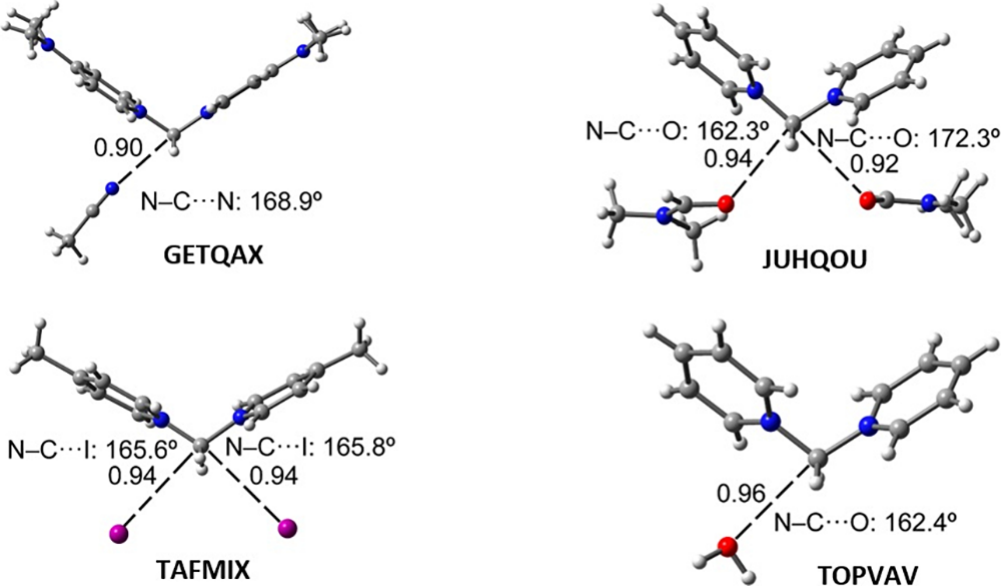
Partial views of the CSD codes GETQAX, JUHQOU, TAFMIX, and TOPVAV with indication of the TtBs as dashed lines. Nc values and angles are indicated. See Table 1 for the binding energies.

Finally, Table 1 gathers the interaction energies computed for TtB complexes **1**–**4** and for similar assemblies retrieved from the Cambridge Structural Database (CSD, see Figure 6 for geometric features). As expected, when donors of electron density are neutral, binding energies are significantly smaller (in absolute value) than when they are anionic.

**Table 1:** Interaction Energies (*E*, Kcal/Mol) and C(sp^3^)···Donor Atom Distances (R, pm) of Adducts **1**–**4** and Analogous Systems from CSD^a^

compound	donor moiety	*E*	*R*
**1**	**I**^–^	–217.1	363
**2**	Pd(C**N**)_4_]^2–^	–279.2	316, 327
**3**	Pt(C**N**)_4_]^2–^	–281.9	317, 325
**4**	**Br**^–^	–218.9	347
**GETQAX**	CH_3_C**N**	–16.9	299
**JUHQOU**	HC(**O**)N(CH_3_)_2_	–44.6	300, 307
**TAFMIX**	**I**^–^	–212.6	365, 367
**TOPVAV**	H_2_**O**	–14.1	313
**YOWMOM**	H_2_**O**	–10.8	317

a Donor atom is in bold.

In the case of TAFMIX,^24^ where iodide is the electron donor, the strength of the TtBs is slightly weaker (−212.6 kcal/mol) than that of compound **1** (−217.1 kcal/mol), likely due to the slightly donating nature of the methyl group attached to the pyridinium ring.

## Conclusions

In conclusion, experimental and theoretical results consistently prove that if two strong electron-withdrawing groups are bonded to a methylene, its electrophilic character is boosted to the point that carbon functions as a bidentate TtB donor. The TtB acceptor can be a neutral or anionic site. The resulting C···nucleophile interactions affect the adopted crystal packing and may thus be useful for controlling the solid architecture of systems as challenging as catenanes and rotaxanes.^9^ This finding is consistent with prior computations that suggest a similar sort of dual binding scenario in the case of tetrel atoms heavier than C, but without benefit of charge assistance.^29,30^

## Supporting Information Available

Synthesis and characterization of compounds studied in this work, CSD searches, and additional structural figures (PDF)
