# Supplementary material for: Geminal Charge-Assisted Tetrel Bonds in Bis-Pyridinium Methylene Salts
Source: Cryst Growth Des. 2023 Feb 14;23(3):1898–902. doi: 10.1021/acs.cgd.2c01386 (PMC10324100; doi:10.1021/acs.cgd.2c01386)
Supplement: Supplementary file 1 — cg2c01386_si_001.pdf [file cg2c01386_si_001.pdf]

Geminal charge assisted tetrel bonds in bis-pyridinium methylene salts

Miriam Calabrese,[a] Andrea Pizzi,[a] Andrea Daolio,[a] Maurizio Ursini,[a] Antonio Frontera,[b] Nicola Demitri,[c] Carsten Lenczyk,[d] Jakub Wojciechowski[e] and Giuseppe Resnati*[a]

ELECTRONIC SUPPLEMENTARY INFORMATION

a NFMLab, Department of Chemistry, Materials, and Chemical Engineering "Giulio Natta" Politecnico di Milano via L. Mancinelli 7; I-20131 Milano, Italy. e-Mail: giuseppe.resnati@polimi.it

b Department of Chemistry Universitat de les Illes Balears Crta. de Valldemossa km 7.5, 07122 Palma de Mallorca (Baleares), Spain.

c Elettra – Sincrotrone Trieste, S.S. 14 Km 163.5 in Area Science Park, 34149 Basovizza – Trieste, Italy.

d Bruker AXS GmbH, Oestliche Rheinbrueckenstr. 49, 76187 Karlsruhe, Germany.

e. Rigaku Europe SE, Hugenottenallee 167. 63263 Neu-Isenburg, Germany.

Table of Content:

S1. Materials and Methods……………………………………………………………………………..3

S.1.1 Materials………………………………………………………………………………….3

S.1.2 Characterization of the compounds………………………………………………………3

S2. Crystallographic Details…………………………………………………………………………5

S.2.1 General Remarks………………………………………………………………………..5

S.2.2 Crystallographic details and Figures of compunds 1-4……………………………….6

S3. CSD Surveys……………………………………………………………..…………………14

S4. Computational Details………………………………………………………………………15

S5. References………………………………………………………………………………………..16

S1. Materials and Methods.

S.1.1 Materials

All the compounds were acquired from commercial suppliers (Sigma-Aldirch, TCI America) and used without further purification.

S.1.2 Characterization of the compounds

General Remarks.

^{1}H and ^{13}C NMR spectra were recorded at ambient temperature on Nuclear Magnetic Resonance Spectrometer AVANCE III, Bruker-BioSpin. All the chemical shifts are given in ppm and the Js in Hz. *D_2O* was used as solvent in NMR spectra. FT-IR spectra were obtained using a Nicolet Nexus FT-IR spectrometer equipped with UATR unit.

*Synthesis of 1,1'-methylene bis(pyridin-1-ium) iodide (**1**)*

An excess of pyridine (65 mL) was added to a solution of diiodomethane (0.2 mmol) in acetonitrile (5 mL). The solution was stirred under reflux overnight. The reaction mixture was cooled to room temperature, the formed amorphous precipitate was filtered, washed with acetonitrile, and then recrystallized in water. FT-IR (selected peaks, cm^{-1}) 3062, 1634, 1490, 1182, 830, 672, 561. ^{1}H NMR (400 MHz, D_2O) δ 7.43 (s, 2H), 8.29 (t, 4H), 8.82 (t, 2H), 9.31 (d, 4H). ^{13}C NMR (101 MHz, D_2O) δ 150.2 (NCCC), 145.1 (NC), 129.0 (NCC), 78.2 (CH_2).

Synthesis of 1,1'-methylene bis(pyridin-1-ium) dichloride

Dichlorometate (5 mL) and pyridine (20 mL) were mixed. The resulting solution was stirred under reflux for 48 hours. The reaction mixture was cooled to room temperature, the formed amorphous precipitate was filtered, washed with acetonitrile and diethyl ether, and vacuum dried. FT-IR (selected peaks, cm^{-1}) 3027, 2874, 1632, 1479. ^{1}H NMR (400 MHz, D_2O) δ 7.41 (s, 2H), 8.28 (t, 4H), 8.82 (t, 2H), 9.30 (d, 4H) ^{13}C NMR (101 MHz, D_2O) δ 150.0 (NCCC), 145.2 (NC), 129.6 (NCC), 78.1 (CH_2).

*Synthesis of 1,1'-methylene bis(pyridin-1-ium) tetracyanidopalladate (**2**)*

0.02 mmol of 1,1'-methylene bis(pyridin-1-ium) dichloride were added to a clear borosilicate vial containing a solution of potassium tetracyanidopalladate (0.04 mmol) in methanol (1.5 mL). An amorphous powder immediately precipitated, it was filtered off and recrystallized in water. FT-IR (selected peaks, cm^{-1}) 3062, 2124, 1634, 1490, 1182, 830, 672, 561. ^{1}H NMR (400 MHz, D_2O) δ 7.45 (s, 2H), 8.28 (t, 4H), 8.80 (t, 2H), 9.29 (d, 4H). ^{13}C NMR (101 MHz, D_2O) δ 150.3 (NCCC), 145.0 (NC), 129.6 (NCC), 124.5 (Pd-CN), 78.1 (CH_2).

*Synthesis of 1,1’-methylene bis(pyridin-1-ium) tetracyanidoplatinate (**3**)*

0.02 mmol of 1,1’-methylene bis(pyridin-1-ium) dichloride were added to a clear borosilicate vial containing a solution of potassium tetracyanidoplatinate (0.04 mmol) in methanol (1.5 mL). An amorphous powder immediately precipitated, it was filtered off and recrystallized in water. FT-IR (selected peaks, cm^{-1}) 3062, 2124, 1634, 1490, 1182, 830, 672, 561. ^{1}H NMR (400 MHz, D_2O) δ 7.27 (s, 2H), 8.18 (t, 4H), 8.69 (t, 2H), 9.18 (d, 4H). ^{13}C NMR (101 MHz, D_2O) δ 150.2 (NCC$\underline{C}$), 145.2 (N$\underline{C}$), 129.8 (NC$\underline{C}$), 124.6 (Pt-$\underline{C}$N), 78.0 ($\underline{C}H_2$).

Synthesis of 1,1’-methylene bis(picolin-1-ium) bromide (**4**)

An excess of dibromomethane (3.5 mL) was added to a solution of 4-picoline (1 mL). The solution was refluxed under stirring overnight. The reaction mixture was cooled to room temperature, the formed amorphous precipitate was filtered, washed with acetonitrile, and then recrystallized in methanol. FT-IR (selected peaks, cm^{-1}) 3389, 3021, 1635, 1478, 1186, 1031, 829, 770, 577. ^{1}H NMR (400 MHz, D_2O) δ 2.70 (s, 6H), 7.20 (s, 2H), 8.06 (d, 4H), 9.02 (d, 4H). ^{13}C NMR (101 MHz, D_2O) δ 165.5 (NCC$\underline{C}$), 143.6 (N$\underline{C}$), 129.9 (NC$\underline{C}$), 76.8 ($\underline{C}H_2$), 22.1 ($\underline{C}H_3$).

S2. Crystallographic Details

S.2.1. General Remarks:

For compound 1: Data collections were performed at the XRD2 beamline of the Elettra Synchrotron, Trieste (Italy).[1] The crystals were dipped in NHV oil (Jena Bioscience, Jena, Germany) and mounted on the goniometer head with kapton loops (MiTeGen, Ithaca, USA). Complete datasets were collected at 100 K (nitrogen stream supplied through an Oxford Cryostream 700) through the rotating crystal method. Data were acquired using monochromatic wavelength of 0.620 Å on Pilatus hybrid-pixel area detectors (DECTRIS Ltd., Baden-Daettwil, Switzerland). The diffraction data were indexed, integrated and scaled using XDS.[2] The structure was solved by the dual space algorithm implemented in the SHELXT code.[3] Fourier analysis and refinement were performed by the full-matrix least-squares methods based on F^2 implemented in SHELXL (Version 2018/3).[4] The Coot program was used for modeling.[5] Anisotropic thermal motion refinement have been used for all atoms. Hydrogen atoms were included at calculated positions with isotropic $U_{factors} = 1.2 \bullet U_{eq}$ or $Uf_{actors} = 1.5 \bullet U_{eq}$ for solvent hydrogens (U_{eq} being the equivalent isotropic thermal factor of the bonded non hydrogen atom). Pictures were prepared using Ortep-3[6] and CCDC Mercury[7] software.

For compound 2: The single crystal data were collected at 100 K (nitrogen stream supplied through an Oxford Cryostream 1000) using a Bruker D8 VENTURE diffractometer, equipped with a PHOTON III photon-counting detector. Unit cell refinement and data reduction were performed using SHELXL-2018/3.[4]

For compound 3: The single crystal data were collected at 100 K (nitrogen stream supplied through an Oxford Cryostream 1000) using a XtaLAB Synergy diffractometer, equipped with a HyPix detector. Unit cell refinement and data reduction were performed using CrysAlisPro 1.171.41.98a.

For compound 4: The single crystal data of the compounds were collected at room temperature using a Bruker SMART APEX II CCD area detector diffractometer. Data collection, unit cell refinement and data reduction were performed using Bruker SAINT. Structures were solved by direct methods using SHELXT[3] and refined by full-matrix least-squares on F^2 with anisotropic displacement parameters for the non-H atoms using SHELXL-2016/6[4]. Absorption correction was performed based on multi-scan procedure using SADABS. Structure analysis was aided by use of the programs PLATON.[8] The hydrogen atoms were calculated in ideal positions with isotropic displacement parameters set to $1.2 \bullet U_{eq}$ of the attached atom.

S.2.2 Crystallographic details and Figures of compounds 1-4.

Table S.1 Crystal data and structure refinement for 1,1'-methylene bis(pyridin-1-ium) iodide (**1**).

Empirical formula	$C_{11}H_{14}I_2N_2O$
Formula weight	444.04
Temperature/K	100K
Crystal system	Orthorhombic
Space group	Fdd2
a/Å	17.381(4)
b/Å	19.484(4)
c/Å	8.523(2)
α/°	90
β/°	90
γ/°	90
Volume/Å^3	2886.3(10)
Z	8
ρ_{calc}g/cm^3	2.044
μ/mm^{-1}	2.967
F(000)	1664.0
Crystal size/mm^3	0.1 × 0.05 × 0.02
Radiation	Synchrotron (λ = 0.620)
2Θ range for data collection/°	4.988 to 59.952
Index ranges	$-28 \leq h \leq 28$, $-31 \leq k \leq 31$, $-13 \leq l \leq 13$
Reflections collected	16928
Independent reflections	3074 [R_{int} = 0.0645, R_{sigma} = 0.0441]
Data/restraints/parameters	3074/3/66
Goodness-of-fit on F^2	0.994
Final R indexes [I>=2σ (I)]	R_1 = 0.0402, wR_2 = 0.0927
Final R indexes [all data]	R_1 = 0.0626, wR_2 = 0.1013
Largest diff. peak/hole / e Å^{-3}	1.95/-1.55
Flack parameter	0.02(4)
CCDC number	2174457

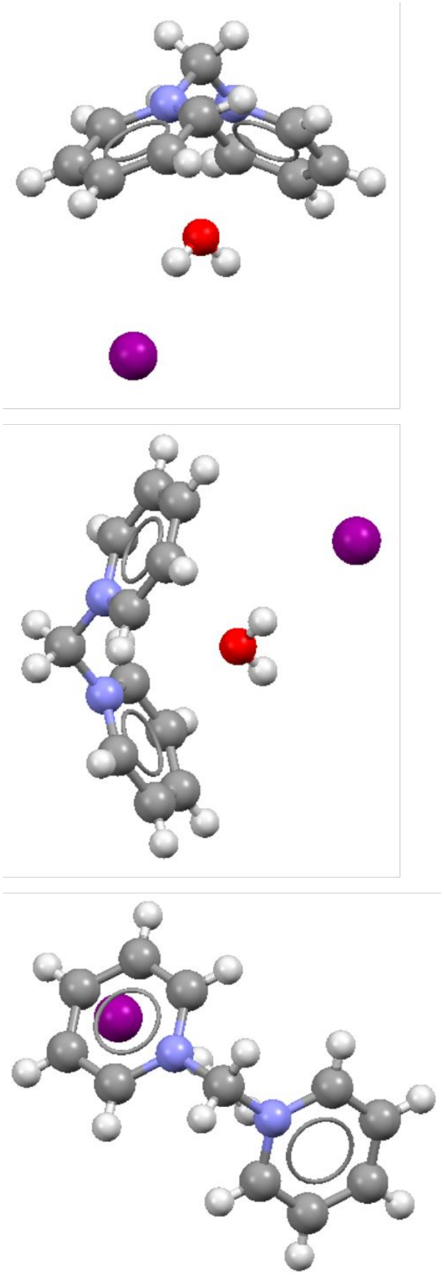

Figure S.1 Unit cell content of **1** along the three crystal axes *a* (top), *b* (mid), *c* (bottom). Color code grey carbon, whitish hydrogen, indigo nitrogen, red oxygen, purple iodine.

Table S.2 Crystal data and structure refinement for 1,1'-methylene bis(pyridin-1-ium) tetracyanidopalladate (**2**).

Empirical formula	$C_{15}H_{12}N_6Pd$
Formula weight	382.71
Temperature/K	100(2)
Crystal system	Monoclinic
Space group	$P2_1/c$
a/Å	13.2862(9)
b/Å	6.7076(5)
c/Å	18.0470(12)
α/°	90
β/°	108.697(2)
γ/°	90
Volume/Å^3	1523.45(18)
Z	4
ρ_{calc}g/cm^3	1.669
μ/mm^{-1}	9.871
F(000)	760.0
Crystal size/mm^3	0.212 × 0.185 × 0.112
Radiation	CuKα (λ = 1.54178)
2Θ range for data collection/°	7.024 to 148.776
Index ranges	$-16 \le h \le 15$, $-8 \le k \le 8$, $-22 \le l \le 22$
Reflections collected	38474
Independent reflections	3098 [R_{int} = 0.0290, R_{sigma} = 0.0169]
Data/restraints/parameters	3098/0/212
Goodness-of-fit on F^2	1.267
Final R indexes [I>=2σ (I)]	R_1 = 0.0266, wR_2 = 0.0769
Final R indexes [all data]	R_1 = 0.0270, wR_2 = 0.0773
Largest diff. peak/hole / e Å^{-3}	0.56/-0.57
CCDC number	2171064

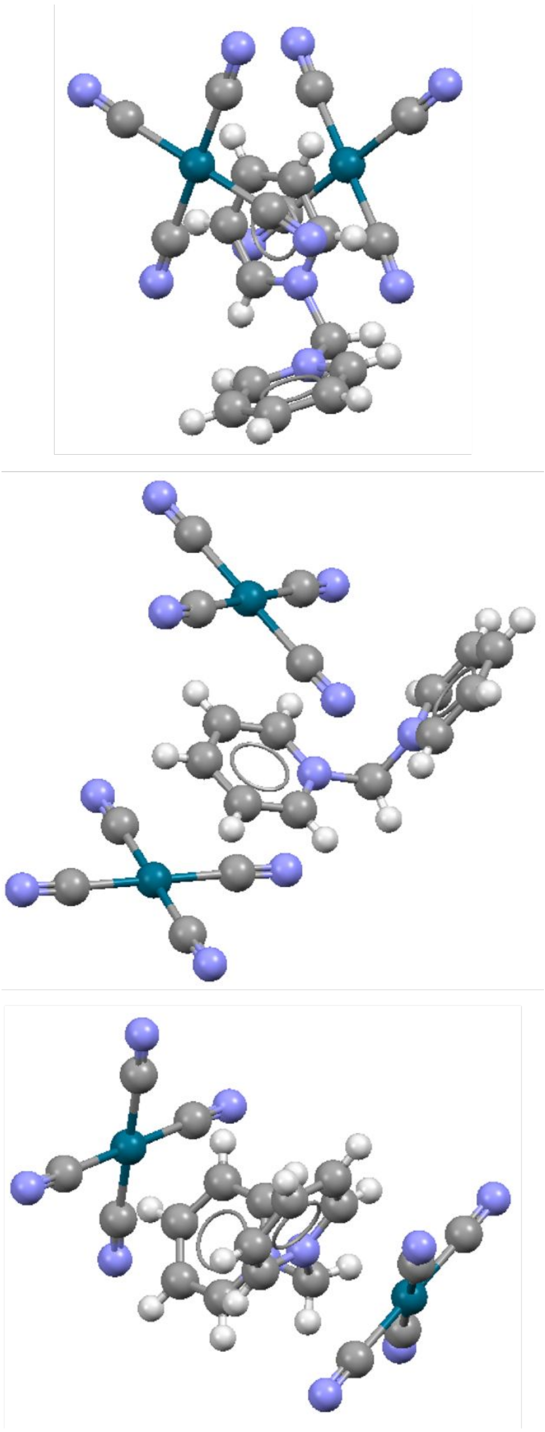

Figure S.2 Unit cell content of **2** along the three crystal axes *a* (top), *b* (mid), *c* (bottom). Color code grey carbon, whitish hydrogen, indigo nitrogen, teal palladium.

Table S.3 Crystal data and structure refinement for 1,1'-methylene bis(pyridin-1-ium) tetracyanidoplatinate (**3**).

Empirical formula	$C_{60}H_{48}N_{24}Pt_4$
Formula weight	1885.58
Temperature/K	100.00(11)
Crystal system	Monoclinic
Space group	$P2_1/n$
a/Å	13.3042(2)
b/Å	6.71230(10)
c/Å	25.6217(5)
α/°	90
β/°	138.273(3)
γ/°	90
Volume/Å^3	1522.89(7)
Z	1
ρ_{calc}g/cm^3	2.056
μ/mm^{-1}	17.281
F(000)	888.0
Crystal size/mm^3	0.184 × 0.148 × 0.084
Radiation	Cu Kα (λ = 1.54184)
2Θ range for data collection/°	7.02 to 162.05
Index ranges	-16 ≤ h ≤ 17, -8 ≤ k ≤ 8, -32 ≤ l ≤ 32
Reflections collected	32558
Independent reflections	3349 [R_{int} = 0.0303, R_{sigma} = 0.0139]
Data/restraints/parameters	3349/0/251
Goodness-of-fit on F^2	1.117
Final R indexes [I>=2σ (I)]	R_1 = 0.0187, wR_2 = 0.0543
Final R indexes [all data]	R_1 = 0.0203, wR_2 = 0.0561
Largest diff. peak/hole / e Å^{-3}	0.89/-0.49
CCDC number	2171059

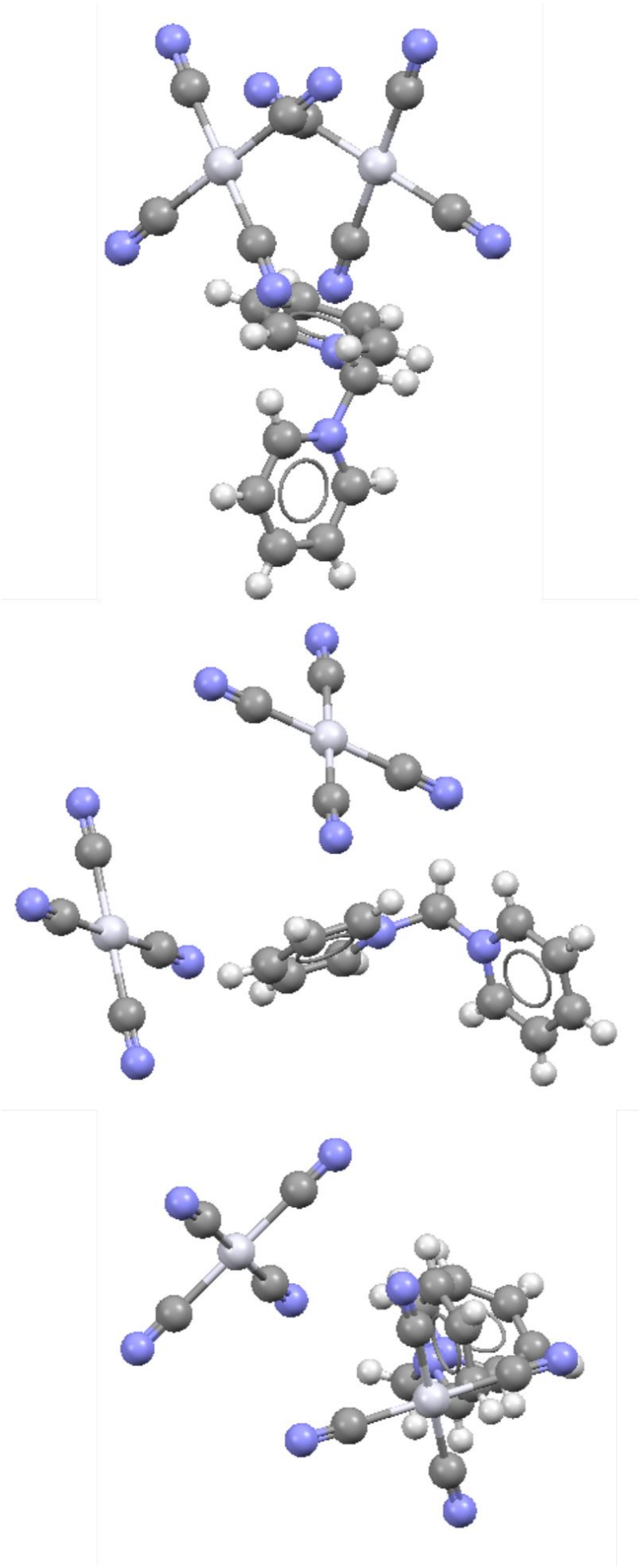

Figure S.3 Unit cell content of **3** along the three crystal axes; top *a*, middle *b*, bottom *c*. Color code grey carbon, whitish hydrogen, indigo nitrogen, light grey platinum.

Table S.4 Crystal data and structure refinement for 1,1'-methylene bis(pyridin-1-ium) bromide (4).

Empirical formula	$C_{6.5}H_8BrNO_{0.5}$
Formula weight	188.05
Temperature/K	300(2)
Crystal system	Monoclinic
Space group	C2/c
a/Å	18.2338(2)
b/Å	8.3699(2)
c/Å	10.18370(10)
α/°	90
β/°	94.8560(10)
γ/°	90
Volume/Å^3	1548.61(4)
Z	8
ρ_{calc}g/cm^3	1.613
μ/mm^{-1}	6.568
F(000)	744.0
Crystal size/mm^3	0.2 × 0.1 × 0.03
Radiation	CuKα (λ = 1.54184)
2Θ range for data collection/°	9.736 to 153.058
Index ranges	-22 ≤ h ≤ 22, -10 ≤ k ≤ 10, -9 ≤ l ≤ 12
Reflections collected	7177
Independent reflections	1520 [R_{int} = 0.0510, R_{sigma} = 0.0263]
Data/restraints/parameters	1520/0/85
Goodness-of-fit on F^2	1.073
Final R indexes [I>=2σ (I)]	R_1 = 0.0491, wR_2 = 0.1470
Final R indexes [all data]	R_1 = 0.0505, wR_2 = 0.1495
Largest diff. peak/hole / e Å^{-3}	1.22/-0.85
CCDC number	2182848

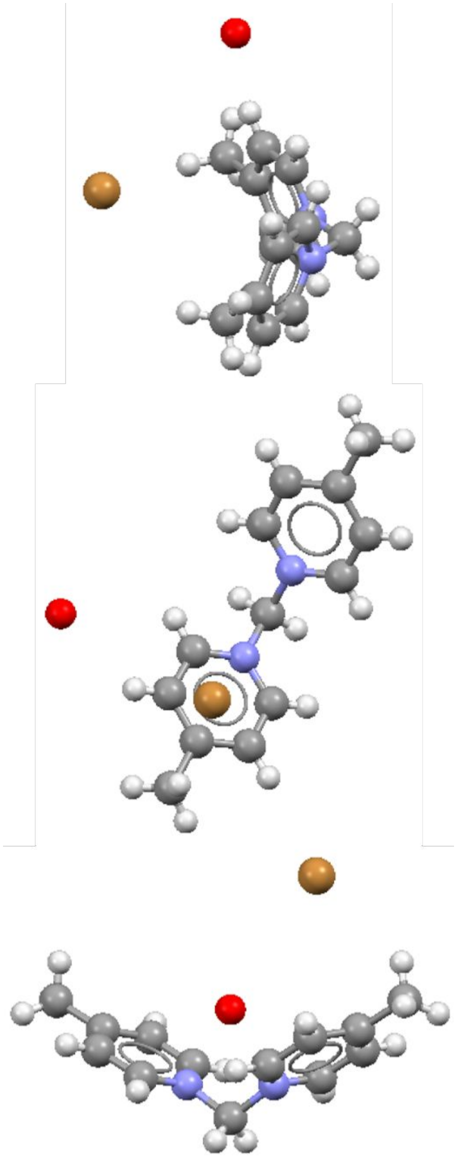

Figure S.4 Unit cell content of **4** along the three crystal axes *a* (top), *b* (mid), *c* (bottom).. Color code grey carbon, whitish hydrogen, indigo nitrogen, red oxygen, gold bromine.

S3. Cambridge Structural Database (CSD) Surveys

Table S.5 Hits in the CSD containing the 1,1'-methylene bis(pyridin-1-ium) moiety. The search is set up with the structure of the 1,1'-methylene bis(pyridin-1-ium) and a positive charge to each nitrogen. In blue, hits displaying a contact between the carbon of the -CH_2- and a nucleophile (N, P, O, S, Se, F, Cl, Br, I considered) shorter than the sum of the respective van der Waals radii and the angle N^+-C···nucleophile between 160 and 180°. According to Batsanov's[9] suggestion, the crystallographic vdW radius of carbon and iodine were set to 170 pm and 210 pm, respectively.

NUQXED02	GIXWUF	NEKTEC	NOYROI	TOPVAV	XAZPET	ZAHGUK
BECSIN	HEVDAN	NEKTIG	NOYRUO	TOPVEZ	XUDLAL	ZAVCEE
BECSOT	HIFHOT	NELBUB	NOYTIE	TOSHIU	YODFIG	ZAVCII
COGKUF	HIFHUZ	NEYJIK	NUQXED	TOSHOA	YODFOM	ZAVCOO
DEHKEG	HIFYEA	NIDCUY	NUQXED01	USEZAU	YOWMOM	ZELZAR
DEHKEG01	HIWWEP	NIDDAF	RACQAN	USEZEY	YUHRIC	ZELZEV
DOVLAD	JAFQEM	NIDDEJ	RAGSUN	UZALEN	YUHROI	ZELZIZ
DOWVAM	JUHQOU	NILVOT	RAGTAU	UZALIR	YUSDIB	ZUYYUN
ENOPED	LEZBAT	NIRWIU	RAHZUX	VABBEG	YUSPEJ	
FOQMAZ	MAYLIH	NOYRIC	TOPTUN	WERPIV	YUSPEJ01	

S4. Computational Details

Computational methods

The energetic features of the adducts analyzed in this work were calculated at the PBE0[10]-D3[11]/def2-TZVP[12] level of theory using either the crystallographic coordinates or fully optimized geometries. This level of theory has been used before[13-15] to analyze similar interactions and it has been proved to provide results similar to high level ab initio methods.[16] The GAUSSIAN-16 program has been used for the energetic calculations and NBO analysis.[17] The basis set superposition error for the calculation of interaction energies has been corrected using the counterpoise method.[18] Molecular electrostatic potential (MEP) surfaces have been computed at the same level of theory and represented using several isovalues of electron density to map the electrostatic potential. The QTAIM analysis[19] has been performed using the AIMAll program at the same level of theory.[20]

In order to assess the nature of interactions in terms of being attractive or repulsive and revealed them in real space, we have used NCIPLOT index, which is a method for plotting non-covalent interaction regions,[21] based on the NCI (Non-Covalent Interaction) visualization index derived from the electronic density.[22]

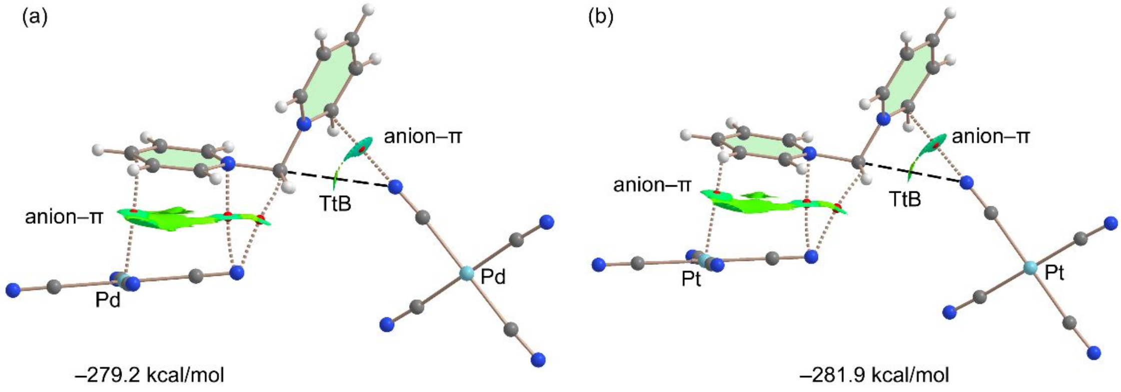

Figure S.5 Combined QTAIM (bond critical points as red spheres and bond paths as dashed lines) and NCIplot representation. The NCIplot settings are: RDG = 0.5, density cut-off 0.04 a.u., colour scale –0.04 a.u. ≤ (sign λ_2)ρ ≤ 0.04 a.u. The interaction energies are also indicated. Only intermolecular interactions are represented.

S5. References

[1] Lausi A., Polentarutti M. et al., The European Physical Journal Plus, **2015** 130(43): 1-8.

[2] Kabsch W. Acta Crystallographica Section D, **2010,** 66(2): 125–132.

[3] Sheldrick, G. M. Acta Crystallographic Section A, **2015,** 71: 3-8.

[4] Sheldrick, G. M. Acta Crystallographica Section C, **2015**, 71: 3-8.

[5] Emsley, P., Lohkamp B., et al., Acta Crystallographica Section D, **2010,** 66(4): 486-501.

[6] Farrugia, L. Journal of Applied Crystallography, **2012**, 45(4): 849-854.

[7] Macrae, C. F., I. Sovago, et al. "Mercury 4.0: from visualization to analysis, design and prediction" Journal of Applied Crystallography, **2020,** 53(1): 226-235.

[8] Spek, A. L. Structure Validation in Chemical Crystallography. *Acta Crystallogr. Sect. D Biol. Crystallogr.* **2009**, *65* (2), 148–155.

[9] Batsanov, S. S. Van Der Waals Radii of Elements. *Inorg. Mater.* **2001**, *37* (9), 871–885.

[10] C. Adamo, V. Barone, *J. Chem. Phys*. **1999**, *110*, 6158-6170.

[11] F. Weigend, *Phys. Chem. Chem. Phys*. **2006**, *8*, 1057-1065.

[12] S. Grimme, J. Antony, S. Ehrlich, H. Krieg, *J. Chem. Phys*. **2010**, *132*, 154104.

[13] M. J. Frisch, G. W. Trucks, H. B. Schlegel, G. E. Scuseria, M. A. Robb, J. R. Cheeseman, G. Scalmani, V. Barone, G. A. Petersson, H. Nakatsuji, X. Li, M. Caricato, A. V. Marenich, J. Bloino, B. G. Janesko, R. Gomperts, B. Mennucci, H. P. Hratchian, J. V. Ortiz, A. F. Izmaylov, J. L. Sonnenberg, Williams, F. Ding, F. Lipparini, F. Egidi, J. Goings, B. Peng, A. Petrone, T. Henderson, D. Ranasinghe, V. G. Zakrzewski, J. Gao, N. Rega, G. Zheng, W. Liang, M. Hada, M. Ehara, K. Toyota, R. Fukuda, J. Hasegawa, M. Ishida, T. Nakajima, Y. Honda, O. Kitao, H. Nakai, T. Vreven, K. Throssell, J. A. Montgomery Jr., J. E. Peralta, F. Ogliaro, M. J. Bearpark, J. J. Heyd, E. N. Brothers, K. N. Kudin, V. N. Staroverov, T. A. Keith, R. Kobayashi, J. Normand, K. Raghavachari, A. P. Rendell, J. C. Burant, S. S. Iyengar, J. Tomasi, M. Cossi, J. M. Millam, M. Klene, C. Adamo, R. Cammi, J. W. Ochterski, R. L. Martin, K. Morokuma, O. Farkas, J. B. Foresman, D. J. Fox, Wallingford, CT, **2016**.

[14] S. F. Boys, F. Bernardi, *Mol. Phys*. **1970**, *19*, 553-566.

[15] R. F. W. Bader, *Chem. Rev*. **1991**, *91*, 893-928.

[16] T. A. Keith, TK Gristmill Software, OverlandPark KS, USA **2019**.

[16] S. J. Grabowski, *J. Mol. Model.* **2013,** *19*, 4713–4721

[17] A. Daolio, A. Pizzi, G. Terraneo, A. Frontera, G. Resnati, A. Daolio, A. Pizzi, G. Terraneo, G. Resnati, A. Frontera, *ChemPhysChem* **2021**, *22*, 2281–2285.

[18] A. Daolio, A. Pizzi, M. Calabrese, G. Terraneo, S. Bordignon, A. Frontera, G. Resnati, *Angew. Chemie - Int. Ed.* **2021**, *60*, 20723–20727.

[19] A. Daolio, A. Pizzi, G. Terraneo, M. Ursini, A. Frontera, G. Resnati, *Angew. Chemie Int. Ed.* **2021**, *60*, 14385–14389.

[20] B. Mallada, A. Gallardo, M. Lamanec, B. de la Torre, V. Špirko, P. Hobza, P. Jelinek, *Science*, **2021**, *374*, 863-867.

[21] J. Contreras-García, E. R. Johnson, S. Keinan, R. Chaudret, J.-P. Piquemal, D. N. Beratan, W.Yang, *J. Chem. Theory Comput.*, **2011**, *7*, 625-632.

[22] E. R. Johnson, S. Keinan, P. Mori-Sánchez, J. Contreras-García, A. J. Cohen, W. Yang, *J. Am. Chem. Soc.*, **2010**, *132*, 6498–6506.
